# Optimising PLGA-PEG Nanoparticle Size and Distribution for Enhanced Drug Targeting to the Inflamed Intestinal Barrier

**DOI:** 10.3390/pharmaceutics12111114

**Published:** 2020-11-19

**Authors:** Lauren J. Mohan, Lauren McDonald, Jacqueline S. Daly, Zebunnissa Ramtoola

**Affiliations:** 1 Division of Biology, Department of Anatomy and Regenerative Medicine, RCSI University of Medicine and Health Sciences, Dublin 2 D02 YN77, Ireland; jdaly@rcsi.ie; 2 School of Pharmacy and Biological Sciences, RCSI University of Medicine and Health Sciences, Dublin 2 D02 YN77, Ireland; laurenmcdonald@rcsi.ie (L.M.); zramtoola@rcsi.ie (Z.R.)

**Keywords:** IBD, nanomedicine, PLGA-PEG, nanoparticles, differential centrifugation, cellular uptake, caco-2 cells

## Abstract

Oral nanomedicines are being investigated as an innovative strategy for targeted drug delivery to treat inflammatory bowel diseases. Preclinical studies have shown that nanoparticles (NPs) can preferentially penetrate inflamed intestinal tissues, allowing for targeted drug delivery. NP size is a critical factor affecting their interaction with the inflamed intestinal barrier and this remains poorly defined. In this study we aimed to assess the impact of NP particle size (PS) and polydispersity (PDI) on cell interaction and uptake in an inflamed epithelial cell model. Using 10, 55 and 100 mg/mL poly(lactic-co-glycolic acid)-polyethylene glycol (PLGA-PEG), NPs of 131, 312 and 630 nm PS, respectively, were formulated by solvent dispersion. NP recovery was optimised by differential centrifugation to yield NPs of decreased and unimodal size distribution. NP-cell interaction was assessed in healthy and inflamed caco-2 cell monolayers. Results show that NP interaction with caco-2 cells increased with increasing PS and PDI and was significantly enhanced in inflamed cells. Trypan blue quenching revealed that a significant proportion of multimodal NPs were primarily membrane bound, while monomodal NPs were internalized within cells. These results are interesting as the PS and PDI of NPs can be optimised to allow targeting of therapeutic agents to the epithelial membrane and/or intracellular targets in the inflamed intestinal epithelium.

## 1. Introduction

Inflammatory bowel diseases (IBD) such as Crohn’s disease and ulcerative colitis are chronic inflammatory disorders of the gastrointestinal (GI) system which affect up to five million people worldwide [1]. Patients need life-long therapy and despite the range of pharmacological treatments available, many still require surgical intervention to manage disease progression. For drug targeting to specific GI tissues such as in IBD, formulations often result in suboptimal drug levels at the desired site of action and adverse off-target effects [2]. Therefore, the development of more advanced strategies for targeted delivery of therapeutics is desirable.

Oral drug delivery is a simple, convenient and non-invasive route of administration and is therefore preferred by clinicians and patients alike [3]. Despite these advantages there are significant obstacles which oral formulations must overcome to achieve appropriate drug delivery and absorption. Many oral formulations exhibit low oral bioavailability due to instability in the GI environment, poor aqueous solubility and membrane permeability [4]. Research in the field of nanomedicine proposes an innovative strategy to enhance oral drug delivery using nanoparticle (NP) delivery vehicles. The advantage of using NPs for drug delivery and targeting is related to their unique physicochemical properties. In particular, their small size, high surface area and easily manipulated surface properties facilitate penetration of tissues and allow for targeted and controlled release of drugs [5,6]. In the context of IBD, Lamprecht et al., established in preclinical models that orally administered particles show enhanced adherence in inflamed colonic tissues compared to healthy tissues [7]. Orally administered NPs have the potential to provide preferential drug targeting to the inflamed GI tissues in IBD.

For GI targeting, NPs must traverse the intestinal barrier which comprises an overlying mucus layer, the intestinal epithelium and the underlying lamina propria. The intestinal epithelium regulates the transport of molecules across the barrier. Transport can occur by a number of routes and is greatly influenced by particle size (PS). Numerous in vitro studies have examined the effect of PS on NP uptake/transport; however, consensus has yet to be reached on the relevant PS limits and mechanisms of uptake across a healthy and an inflamed GI barrier. Additionally, these studies typically assess monodisperse polystyrene latex NPs which are non-biodegradable and therefore have limited use as drug delivery vehicles [8,9,10].

Investigation of polymeric NPs as drug delivery vehicles increased vastly in the last three decades following the Food and Drug Administration’s (FDA) approval of the biodegradable synthetic polymers poly (lactic acid) (PLA), poly (lactic-co-glycolic acid) (PLGA) and poly (glycolic acid) (PGA). Of these polymers, PLGA has been the most widely adopted for NP formulation due to its design flexibility and favourable degradation profile [11,12].

To date, few studies have examined the impact of PS using biodegradable polymeric NPs across the nano size range (1–1000 nm). While studies examining polystyrene NPs typically report that NP uptake is size-dependent, favouring smaller NPs (< 200 nm), studies which apply biodegradable NPs are more ambiguous. McClean et al., reported enhanced interaction of PLGA particles ranging from 630–1680 nm in size in a caco-2 cell model [13]. Similarly, Gaumet et al., found comparable levels of interaction of different sized PLGA NPs in the range of 100–1000 nm in caco-2 cell monolayers [14]. Conversely, Li et al., noted greater interaction/uptake for 50 nm NPs than larger NPs (up to 720 nm) when examining alginate NPs in a similar in vitro model [15]. For effective treatment of IBD, understanding how different sizes of NPs interact with the gastrointestinal barrier is essential in optimising the delivery of agents to specific cell types and sites of disease.

A number of studies have also highlighted the crucial role NP surface properties play in their interactions with biological barriers. Incorporation of hydrophilic polyethylene glycol (PEG) has been demonstrated to enhance the ability of NPs to penetrate the intestinal barrier [16]. Kirby et al., demonstrated that PEGylation of PLGA NPs enhanced uptake of larger NPs over smaller non-PEGylated NPs in an in vitro intestinal barrier model [17]. Larger NPs have the added advantage of greater drug-loading potential and slower release kinetics.

In this study we aimed to prepare and characterise PLGA-PEG NPs of three defined sizes of 100, 300 and 600 nm, using a solvent dispersion method. NPs were optimised for low polydispersity and size uniformity. The interaction and uptake of these size-refined NPs were studied in healthy and inflamed epithelial cell models in caco-2 monolayers using flow cytometry and confocal microscopy to understand their contribution to cell uptake and transport across the GI epithelial barrier.

## 2. Materials and Methods

### 2.1. Materials

PLGA-PEG polymer, Resomer^®^ Select 5050 DLG mPEG 5000 10 wt% PEG, was purchased from Evonik Industries AG (Darmstadt, Germany). Coumarin-6, 98% and Tween^®^80 were obtained from Sigma–Aldrich, (Wicklow, Ireland). Transmission electron microscopy TEM grids, Formvar/Silicon Monoxide, were purchased from Mason Technologies (Dublin, Ireland). HCS CellMask^TM^ Deep Red Stain was obtained from Fisher Scientific (Dublin, Ireland). Inflammatory cytokines interleukin 1 beta (IL-1β), tumour necrosis factor (TNF) and interferon gamma (IFN-γ) were purchased from Biolegend (London, UK). All other solvents and reagents used were of reagent grade and were purchased from Sigma–Aldrich.

### 2.2. Formulation of PLGA-PEG NPs

**Figure 1:**
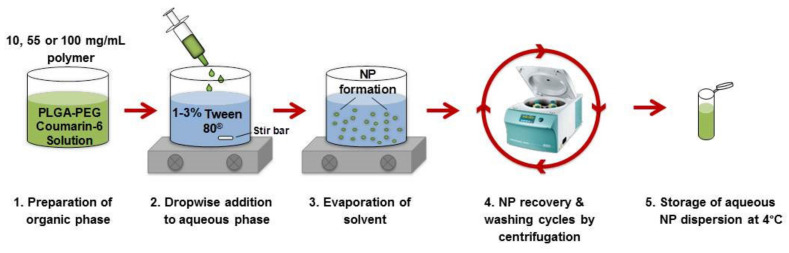
Schematic representation of the solvent dispersion method used to prepare PLGA-PEG NPs.

Fluorescently-labelled PLGA-PEG NPs were formulated using a solvent dispersion method as shown in Figure 1 [11]. Briefly, PLGA-PEG polymer solution in acetone at 10, 55 and 100 mg/mL containing coumarin-6 fluorescent marker at 0.5% *w*/*w* of polymer was added in a drop-wise manner to an aqueous solution containing 1–3% *w*/*v* Tween^®^80. Then, 5 mL of the polymer solutions were added to the 15 mL aqueous phase, giving a solvent to non-solvent ratio of 0.25. The resulting dispersion was stirred for 18 h to allow for evaporation of the solvent. NPs were then recovered via centrifugation (Rotina 35 R centrifuge, Hettich Zentrifugen, Germany) at 15,557 relative centrifugal force (RCF) for 20 min at 4 °C. Pelleted NPs were washed with deionised water and centrifuged. Washed NPs were redispersed in water and stored at 4 °C.

### 2.3. NP Characterisation

The average particle size (PS), polydispersity index (PDI) and zeta potential (ZP) of NPs were measured by dynamic light scattering (DLS) using a Malvern Zetasizer Nano ZS 90 (Malvern Instruments, Worcestershire, UK). All sample measurements were performed at a scattering angle of 173 °C and a temperature of 25 °C. Samples for each NP batch were measured five times and the average value ± the standard deviation reported. Size distribution peaks by intensity were also recorded for each measurement.

NP morphology was observed by transmission electron microscopy (TEM). Aqueously dispersed NPs were added to a formvar grid stabilised with silicon monoxide. Following evaporation of the dispersant, sample grids were viewed using a Hitachi H-7650 electron microscope (Hitachi High-Technologies, Leixlip, Ireland).

### 2.4. Size Distribution Analysis of NPs Using Differential Centrifugation

**Figure 2:**
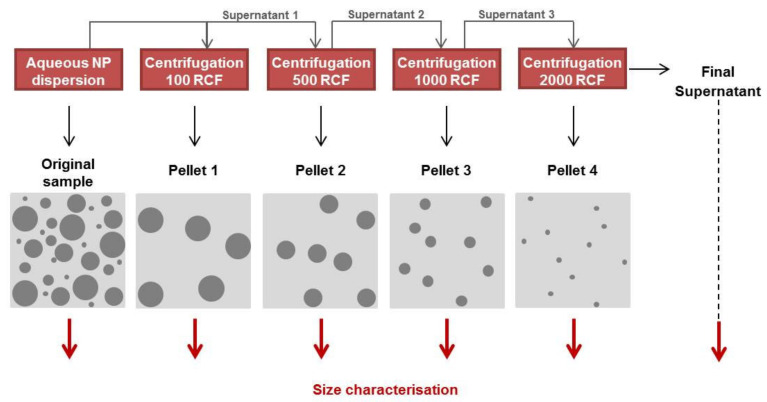
Differential centrifugation of polydisperse PLGA-PEG NPs. Schema of the centrifugation strategy applied to isolate NPs of different PS.

Formulated NPs were dispersed in 7 mL water in a 15 mL conical tube. A centrifugation protocol of increasing speed was used to separate the various size fractions of NPs with high PDI (Figure 2). Samples were first centrifuged at 100 RCF for 20 min and the pelleted sample (pellet 1) and supernatant (supernatant 1) were separated. Supernatant 1 was further centrifuged at 500 RCF for 20 min to obtain pellet 2 and supernatant 2, which were again separated. This process was repeated with further centrifugations at 1000 and 2000 RCF of supernatants 3 and 4, respectively. Pelleted fractions 1–4 were appropriately redispersed in water and analysed by DLS to obtain size distribution peaks along with supernatant 4 (final supernatant).

### 2.5. Cell Culture

Caco-2 cells (ECACC, Sigma–Aldrich), passage number 49–65, were cultured in Dulbecco’s modified eagle medium (DMEM) containing 25 mM glucose supplemented with 10% *v*/*v* heat inactivated foetal bovine serum (FBS), 1% *v*/*v* non-essential amino acids (NEAA), 1% *v*/*v* penicillin-streptomycin and 2 mM L-glutamine. Cells were grown in 75 cm^2^ tissue culture flasks at 37 °C with 5% CO_2_ and 95% relative humidity, changing the culture medium every 2–3 days [18].

### 2.6. In Vitro Interaction of PLGA-PEG NPs With Caco-2 Cells

Cells were seeded in 24-well cell culture plates at 3 × 10^5^ cells per well and grown for 48 h until confluent monolayers formed [19]. For the inflamed cell model, confluent caco-2 cells were exposed to an inflammatory cocktail of IL-1β (25 ng/mL), TNF (50 ng/mL) and IFN-γ (50 ng/mL) for 24 h prior to initiation of NP studies [20]. NPs (1 mg/mL) were dispersed in a 1:1 ratio of sterile DMEM/Hanks balanced salt solution (HBSS) and were applied to the cells and incubated for 2 h at 37 °C. Coumarin-6 released from NPs under the above in vitro conditions was also incubated with cells for 2 h to act as a fluorescence control. After incubation, cells were washed three times with sterile PBS to remove unbound NPs. Cells were fixed and stained with HCS CellMask^TM^ Deep Red Stain and mounted on microscope slides using Fluoroshield^TM^ with DAPI mounting medium for confocal laser scanning microscopy (CLSM). For flow cytometry analysis, cells were harvested using 0.25% *w*/*v* trypsin-EDTA solution, and 10,000 events were recorded/per sample using the Fl-1 channel. To evaluate NP internalisation, cells were incubated with 0.11% *v/v* trypan blue for 1 min to quench the green fluorescent signal from NPs adsorbed to the cell surface and were analysed by flow cytometry as above [21]. Results were reported as the geometric mean of the distribution of cell fluorescence intensity.

### 2.7. Statistical Analysis

Statistical analysis was performed using GraphPad Prism. Where a statistical difference was revealed, a post-hoc test for multiple comparisons was used. A *p* value < 0.05 was considered to be statistically significant, *p* < 0.01 very significant and *p* < 0.001 highly significant.

## 3. Results and Discussion

### 3.1. NP Characterisation

To select formulation parameters, a preliminary study was conducted where PLGA-PEG NPs were prepared by solvent dispersion, and the effect of varying the polymer concentration from 10–100 mg/mL and Tween^®^80 concentration from 1–3% *w*/*v* on NP characteristics was studied using a randomised order of experiments. The physicochemical characteristics of the prepared NPs are outlined in Table 1. All formulations produced particles in the nanoscale range with PS observed in the range of 146–601 nm. PDI for these formulations ranged from near monodisperse at 0.20 to very polydisperse at 0.76. A negative zeta potential was reported for all formulations with values ranging from −27 to −20 mV.

Statistical analysis based on two-way ANOVA revealed that polymer concentration had the most significant effect on PS (*p* < 0.0001), with PS increasing with polymer concentration. This finding confirms previous reports in the literature which identified polymer concentration as the most influential parameter on PS [22,23]. Tween^®^80 concentration was also found to have a significant effect on PS (*p* < 0.05), decreasing PS as the Tween^®^80 concentration increased. Increasing the viscosity of the aqueous phase and reducing surface tension allow for smaller particle formation during solvent dispersion [24].

Polymer concentration and Tween^®^80 concentration both had very statistically significant effects on PDI (*p* < 0.0001). As with PS, PDI increased with polymer concentration and PDI decreased with increasing Tween^®^80 concentrations. Increased amounts of Tween^®^80 in the aqueous phase have been shown to promote hydrodynamic stabilization, therefore preventing aggregations which contribute to larger PDI values [25]. For NPs prepared at 55 mg/mL polymer concentration, those prepared with 1% Tween^®^80 had a PDI significantly higher than the 2% (*p* < 0.05) and 3% (*p* < 0.001) formulations. These findings suggest that Tween^®^80 concentrations of at least 2% may be more appropriate in the preparation of NPs by solvent dispersion.

Tween^®^80 concentration alone had a significant impact on NP ZP (*p* < 0.001). Tween^®^80 is a non-ionic surfactant, which if incorporated in to the NP surface during solvent dispersion could affect the negative ZP typically observed for PLGA-PEG NPs [26]. For 55 mg/mL and 100 mg/mL NPs prepared at 3% Tween^®^80 concentration, more neutral ZP values were recorded. An intermediate concentration of Tween^®^80 (2%) was selected as a fixed formulation parameter and only the polymer concentration was varied in future experiments for the preparation of NPs of 100, 300 and 600 nm.

The physicochemical characteristics of NP batches prepared using selected parameters are summarised in Table 2. Increasing the PLGA-PEG concentration from 10 to 55 and 100 mg/mL resulted in an increase in PS, generating NPs of average PS of 132 nm, 312 nm and 631 nm, respectively. Statistical analysis based on one-way ANOVA revealed that NPs formulated at each polymer concentration were of distinct average PS which were significantly different from each other (*p* < 0.001) Increasing the polymer concentration from 10 to 100 mg/mL also resulted in a significant increase in the PDI of NP samples from 0.15 to 0.68 (*p* < 0.05).

ZP values in the range of −21 to −26 mV were recorded for all NPs prepared. Increasing PLGA-PEG concentration did not lead to statistically significant differences in average ZP (*p* > 0.05). These negative values correspond with those previously recorded for PLGA-PEG NPs [26,27]. ZP values in this range suggest the colloidal stability of NPs in dispersion [28].

**Figure 3:**
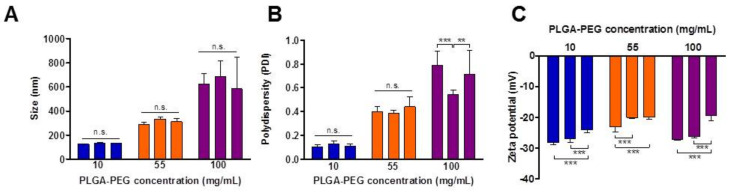
Batch-to-batch reproducibility of PLGA-PEG NP characteristics. Each bar represents an individual batch with values representing the average of *n* = 5 measurements ± standard deviation. (**A**) Average particle size (PS), (**B**) polydispersity index; (PDI) and (**C**) zeta potential (ZP), n.s., not-significant; ** *p* < 0.01; *** *p* < 0.001.

The reproducibility of the NP preparation process using the parameters selected from Table 1, with respect to PS of NPs, was confirmed by one-way ANOVA with Tukey’s post-hoc analysis, comparing NPs formulated at each polymer concentration (Figure 3). The reproducibility of the batches with respect to PDI was demonstrated at the lower polymer concentration of 10 and 55 mg/mL, while at 100 mg/ML, significant differences were observed in PDI values. Statistically significant differences in ZP were noted between batches for all NPs; however, ZP values remained within the stable range of −21 to −26 mV. Given the impact of PS on performance, the reproducibility of NP physicochemical characteristics represents a significant challenge. Methods which generate reproducible NP characteristics are desirable to ensure consistent parameters for pre-clinical studies and at later stages to facilitate production scale-up and translation to the clinic [29].

**Figure 4:**
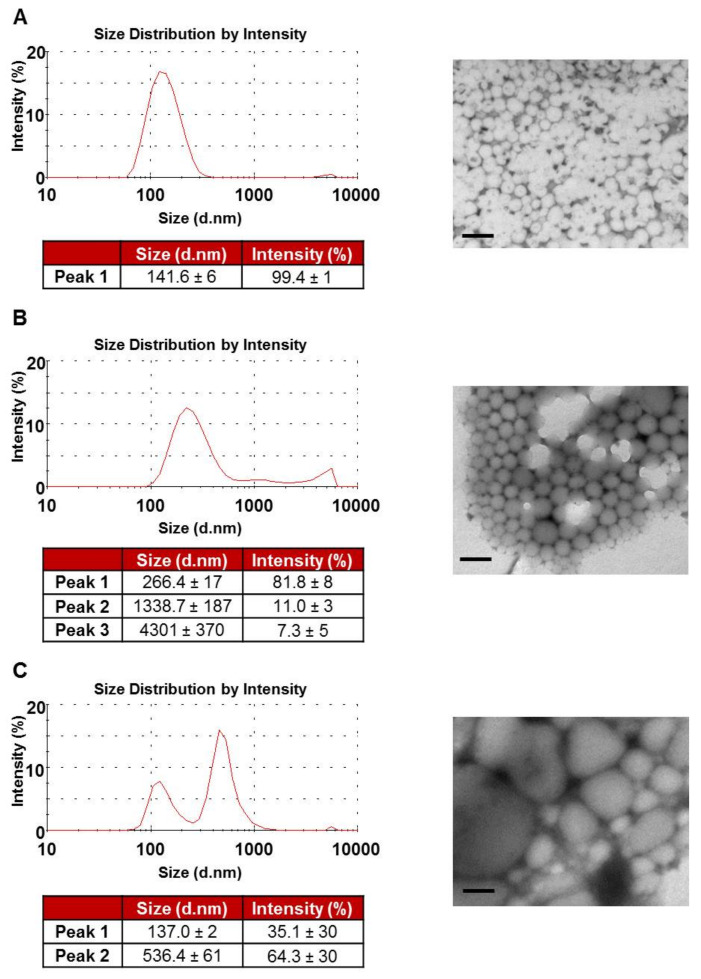
Size distribution analyses of PLGA-PEG NPs. Size distribution peaks by intensity and corresponding transmission electron microscopy micrographs for NPs formulated at (**A**) 10 mg/mL, (**B**) 55 mg/mL and (**C**) 100 mg/mL PLGA-PEG concentration. Scale bar = 300 nm.

The increases observed in PDI as polymer concentration increased were further investigated. The DLS size distribution plots by intensity for NPs prepared at each polymer concentration are presented in Figure 4. At the lowest polymer concentration of 10 mg/mL, a unimodal size distribution was observed with a single peak at 142 nm. NPs prepared at a 55 mg/mL polymer concentration displayed a trimodal size distribution with a primary peak at 266 nm with two minor peaks observed at 1339 nm and 4301 nm, probably related to NP association or aggregates. Interestingly, NPs prepared at the highest polymer concentration of 100 mg/mL displayed a bimodal size distribution with two distinct peaks observed at 137 nm and 536 nm. The PS and size distribution of NPs were confirmed in the TEM shown in Figure 4. In Figure 3A, the average values reported by DLS represent the average hydrodynamic radius, in nm, of NPs and hence do not account for the presence of multiple modes in the NP size population. This is of particular relevance to samples with higher PDI values.

### 3.2. Size Distribution Analysis of NPs Using Differential Centrifugation

**Figure 5:**
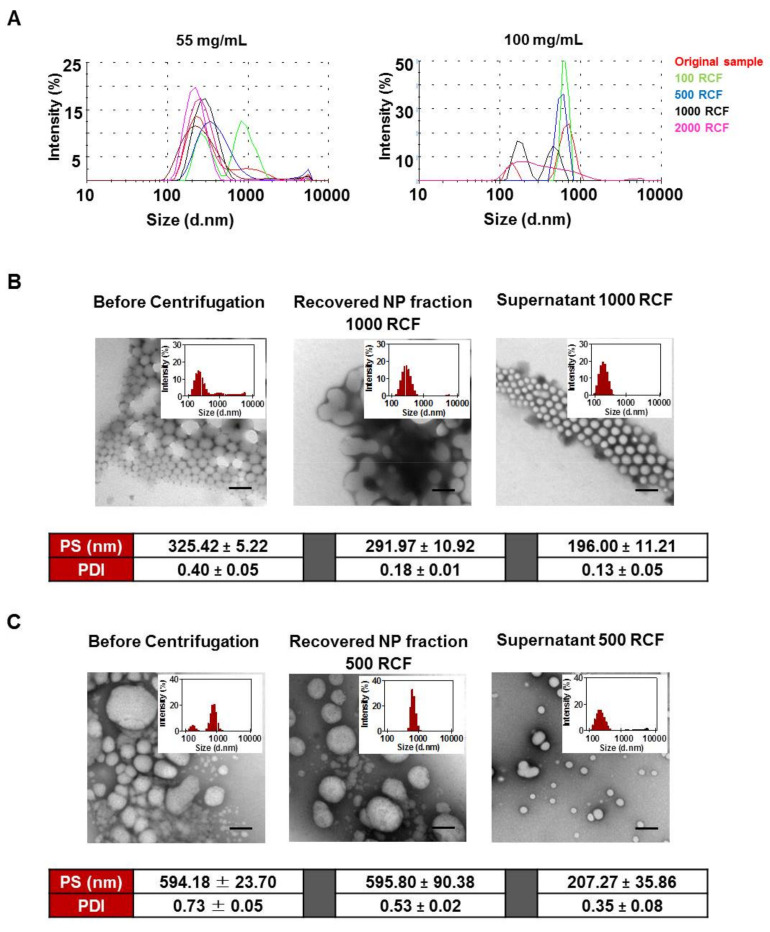
Optimisation of NP recovery to reduce polydispersity. (**A**) Size distribution profiles of pelleted size fractions recovered by differential centrifugation of PLGA-PEG NPs prepared at 55 and 100 mg/mL polymer concentrations. Representative size distributions and TEM images of NPs prepared with (**B**) 55 mg/mL and (**C**) 100 mg/mL PLGA-PEG before and after optimisation of recovery by centrifugation at 1000 RCF and 500 RCF, respectively. Average PS and PDI are included below images ± SD, *n* = 3. Scale bar = 200 nm.

Using the differential centrifugation strategy presented in Figure 2, the size distribution profiles of NPs, prepared at 55 and 100 mg/mL PLGA-PEG and recovered at increasing centrifugation speeds, were analysed (Figure 5A). Optimal recovery speeds were then selected for each NP group. The effect of the optimised recovery strategy on PS and PDI of the NPs (Figure 5B,C) confirms that UM size distribution profiles were achieved for both NP groups. A significant decrease in PS following recovery was noted for NPs prepared at 55 mg/mL (*p* < 0.05) while no significant change in PS was observed for NPs prepared at 100 mg/mL (*p* > 0.05). A significant reduction in PDI following centrifugation was observed for both NP groups. Average PDI decreased from 0.40 to 0.18 and from 0.73 to 0.53 for NPs prepared at 55 mg/mL (*p* < 0.01) and 100 mg/mL (*p* < 0.05), respectively. TEM images confirm the separation of the different sized NP populations by centrifugation with superfine NPs of approx. 200 nm in size present in the remaining supernatant samples (Figure 5B,C).

Currently, filtration methods may be applied to remove larger particles and aggregates in polydisperse NP samples. However superfine NPs remain and their presence may be a contributor when assessing the impact of different NP sizes on cell interaction and uptake studies. Robertson et al., previously demonstrated the use of differential centrifugation as an effective method for separating NPs into discrete size fractions using polymersomes as a polydisperse NP model [30]. In this study the optimisation of NP centrifugal recovery facilitated refining of the size of the NPs formulated at high PLGA-PEG polymer concentrations of 55 and 100 mg/mL, reducing the trimodal and bimodal size distributions and large PDI towards unimodal size distributions and low PDI. These refined NP size distributions were subsequently examined in cell interaction and uptake studies in caco-2 cell monolayers.

### 3.3. Effect of PS and PDI on NP Uptake/Interaction in Caco-2 Cells

**Figure 6:**
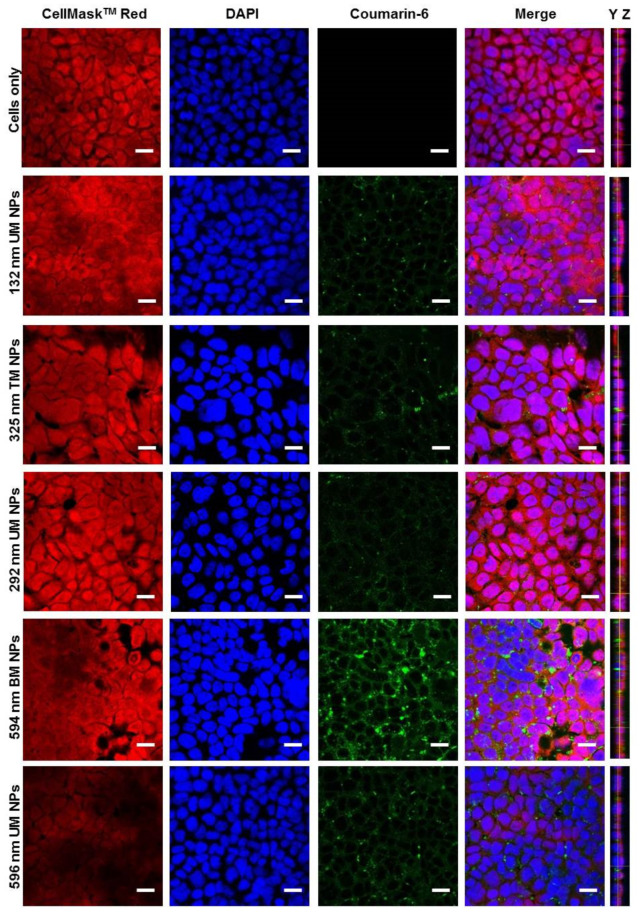
Confocal microscopy analysis of cellular uptake of fluorescently labelled PLGA-PEG NPs in caco-2 cell monolayers after 2 h incubation at 37 °C. Cell nuclei and cytoplasm were stained with DAPI (blue) and HCS CellMaskTM Deep Red, respectively. Images represent the centre of the z-stack. Scale bar = 20 μM. UM, unimodal; TM, trimodal; BM, bimodal.

Unimodal (UM) NPs of 132 nm, 292 nm and 596 nm along with 325 nm trimodal (TM) and 594 nm bimodal (BM) NPs were included in cell studies to assess the effect of NP polydispersity on cell interaction/uptake. Confocal imaging analyses of Caco-2 cell monolayers exposed to NPs for 2 h are presented in Figure 6. An enhanced green fluorescent signal was noted with increasing PS of NPs. YZ orthogonal views of the caco-2 monolayers revealed that NPs were present primarily within the monolayer and on the basolateral side, indicating uptake and transport of NPs through the cell monolayer.

**Figure 7:**
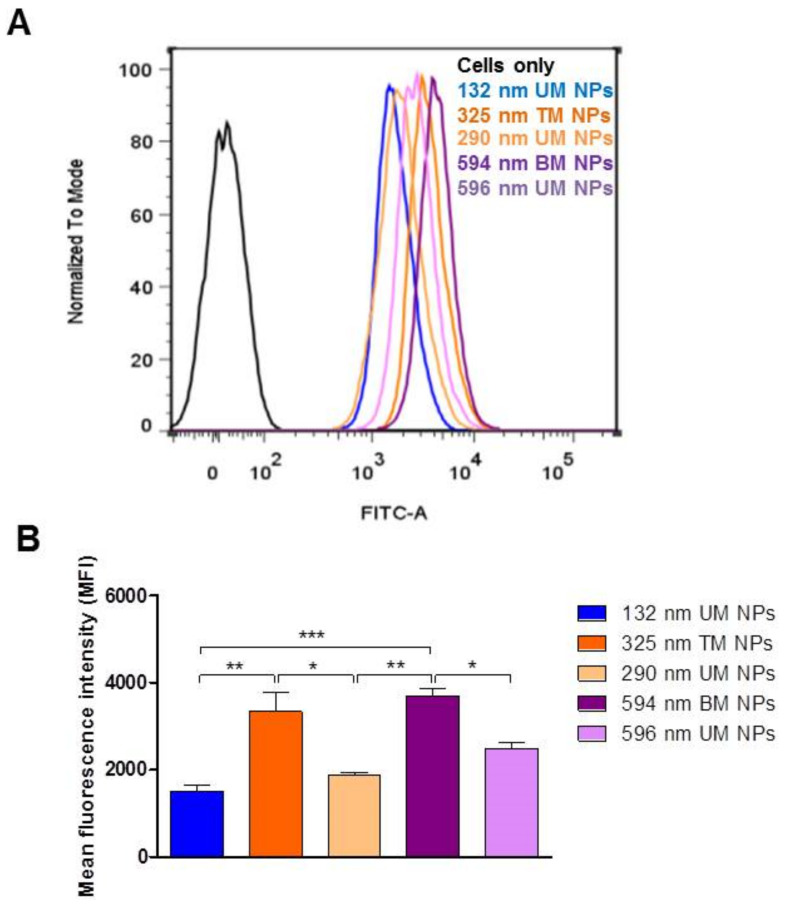
Flow cytometry analysis of caco-2 monolayers following 2 h incubation with NPs at 37 °C. (**A**) Representative fluorescence histograms of % cell count vs. log fluorescence using the FL-1 (green) channel. (**B**) Quantitative analysis of cellular interaction/uptake for each NP size using geometric mean fluorescent intensity calculated using FlowJo software (*n* = 3). * *p* < 0.05; ** *p* < 0.01; *** *p* < 0.001; UM, unimodal; TM, trimodal; BM, bimodal.

Flow cytometry confirmed NP-cell interaction and uptake as was observed by confocal microscopy (Figure 7). Results showed a statistically significant increase in the mean fluorescent intensity (MFI) for all NP-treated cells compared to untreated controls. Cells treated with coumarin-6 released from NPs under in vitro conditions displayed negligible fluorescence confirming that fluorescence observed was indeed associated with NP interaction (Figure S1). Coumarin-6 release from NPs under in vitro conditions was less than 7% (Figure S2).

MFI values were highest for cells exposed to 594 nm BM NPs. A significant decrease in MFI was noted for 292 nm UM NPs and 596 nm UM NPs compared to their polydisperse counterparts, i.e., 325 nm TM NPs and 594 nm BM NPs, respectively (*p* < 0.05). This finding is significant at it suggests that superfine NPs that are present in NP samples of higher PDI can significantly contribute to measured fluorescence and in turn can skew analysis. This highlights the importance of sample monodispersity in size-based examination of NP uptake.

Analysis of UM NPs revealed the highest MFI values for 596 nm UM NPs, suggesting enhanced NP interaction/uptake of larger NPs. NP interaction/uptake for 292 nm UM NPs was higher than for 132 nm UM NPs. Therefore, NP interaction increased with increasing size (596 nm > 292 nm > 132 nm).

Previous literature reports have typically focused on smaller sized NPs (<200 nm) for cell targeting. Interestingly, results from our study suggest that larger NPs in the range of 292–596 nm may also prove useful for such applications. This enhanced interaction could also be attributed to the presence of PEG in the NPs. It has previously been reported that PEGylation enhances the passage of larger NPs across biological barriers [17]. Larger PS can offer the possibility of increased drug-loading and slower drug release which may be advantageous for drug targeting and delivery.

### 3.4. Evaluation of NP Internalisation in Healthy and Inflamed Epithelial Models

**Figure 8:**
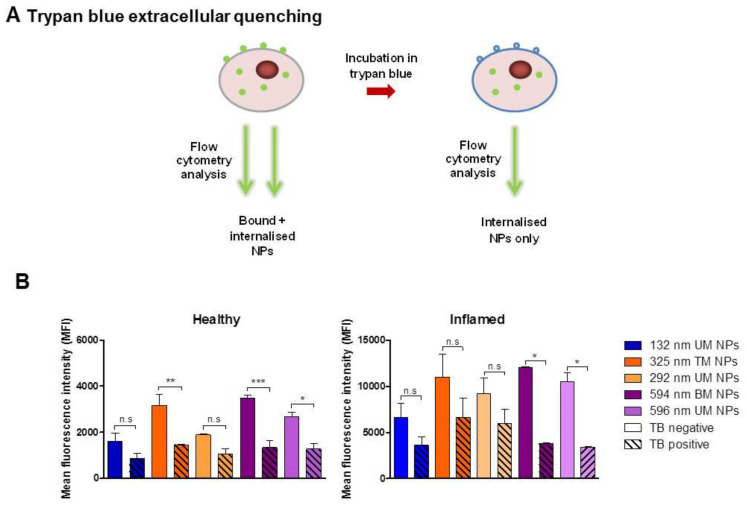
Assessment of NP internalisation in healthy and inflamed caco-2 cells. (**A**) Illustration of trypan blue extracellular quenching of the green fluorescent signal to determine the signal from internalised NPs only. (**B**) Mean fluorescent intensity of caco-2 cells incubated with NPs for 2 h at 37 °C (FL-1 channel) calculated using FlowJo software, (*n* = 3). * *p* < 0.05; ** *p* < 0.01; *** *p* < 0.001; TB, trypan blue; UM, unimodal; TM, trimodal; BM, bimodal.

Figure 8 shows the cellular internalisation of NPs by healthy and inflamed caco-2 cell models as examined by flow cytometry after 2 h incubation at 37 °C. In order to differentiate between membrane-bound and internalised NPs, trypan blue quenching was employed to eliminate the fluorescence of extracellularly-bound NPs (Figure 8A). In healthy cells, trypan blue quenching resulted in a significant decrease in the fluorescent signal for 325 TM NPs, 594 nm BM NPs and 596 nm UM NPs, suggesting the majority of these NPs are extracellularly bound (Figure 8B). For unimodal NPs of 132 nm and 292 nm in size, there was no significant reduction in MFI, suggesting that NPs of these sizes are primarily internalised. These findings suggest that in healthy tissues unimodal NPs of up to 292 nm may be more effective for intracellular targeting of cells, while NPs of larger sizes (325–596 nm) can be employed in targeting the epithelial cell membrane.

Similarly in the inflamed cell model, 594 nm BM NPs and 596 nm UM NPs had a significant proportion of membrane-bound NPs; however, 132 nm UM NPs, 292 nm UM NPs and 325 nm TM NPs were primarily internalised. NP uptake/association was significantly enhanced for all NP sizes in the inflamed model compared to the healthy one (*p* < 0.001). This finding is significant as it further suggests that inflamed tissues can be more effectively targeted by NPs than healthy tissues. Additionally, in the inflamed cells a significant increase in the internalised NP signal for 325 nm TM NPs and 292 nm UM NPs was noted compared to healthy cells (*p* < 0.01, *p* < 0.05, respectively).

NP internalisation in non-phagocytic cells can occur by a number of endocytic mechanisms including clathrin-mediated endocytosis, caveolae-mediated endocytosis and macropinocytosis. Zhou et al., previously reported enhanced uptake of 200 nm Fe_3_ O_4_ NPs by clathrin-mediated mechanisms in an inflamed caco-2 cell model. Inflamed cells were found to express three times as much clathrin as their healthy counterparts [31]. It is known in IBD that inflammatory cytokines such as IL-1β, TNF and IFN-γ cause dysregulation of endocytic mechanisms leading to enhanced uptake of tight junction proteins and bacteria which contribute to barrier disruption and disease pathogenesis [32,33].

## 4. Conclusions

In this study, PLGA-PEG NPs of three sizes were prepared by solvent dispersion using selected formulation parameters. Increasing polymer concentration increased PS, resulting in NPs of 131, 312 and 630 nm. PDI was also found to increase with PS. Following size distribution analysis by differential centrifugation, recovery of NPs was optimised to obtain unimodal NPs with low PDI. Flow cytometry and confocal microscopy results of unimodal and multimodal NP interaction with caco-2 cells highlighted the impact NP PDI can have on cell uptake. NP targeting potential, assessed in healthy and inflamed caco-2 cell models, showed that NP interaction increased with increasing PS of 132 nm to 594 nm. Interaction of NPs was significantly enhanced in the inflamed model compared to the healthy cells. Trypan blue quenching revealed that in healthy cells, unimodal NPs up to 292 nm were primarily internalised while larger multimodal NPs were primarily membrane-bound. Inflamed cells showed enhanced internalisation of trimodal NPs of larger size up to 325 nm, which could be attributed to alterations in endocytic mechanisms which occur during inflammation. Further investigation of specific mechanisms of NP uptake in the inflamed intestinal barrier is warranted. The findings in this study are significant as they suggest that PLGA-PEG NPs can be an effective strategy for targeting therapeutic agents to the inflamed IBD tissues and that NP size and PDI are important factors for consideration in optimising their targeting potential.

## Figures and Tables

**Table 1:** Physicochemical characteristics of PLGA-PEG NPs prepared by solvent dispersion where both the PLGA-PEG concentration and Tween^®^80 concentration were varied.

Experiment No.	PLGA-PEG (mg/mL)	Tween 80^®^ (% *w*/*v*)	PS ± SD (nm)	PDI ± SD	ZP ± SD (mV)
1	100	1	600.86 ± 192.94 ^a^	0.76 ± 0.11 ^a^	−22.62 ± 0.49 ^b^
2	10	3	145.76 ± 4.60 ^e^	0.20 ± 0.02 ^d^	−23.40 ± 1.39 ^e^
3	10	2	145.50 ± 3.53 ^d,e^	0.28 ± 0.03 ^d^	−22.82 ± 2.72 ^e^
4	100	3	417.42 ± 237.27 ^b^	0.63 ± 0.19 ^a^	−20.00 ± 0.90 ^c^
5	10	1	167.72 ± 8.13 ^d^	0.32 ± 0.04 ^d^	−22.32 ± 2.67 ^e^
6	55	1	293.48 ± 27.38 ^c^	0.51 ± 0.03 ^b^	−23.18 ± 0.51 ^d^
7	55	3	177.66 ± 5.22 ^c^	0.29 ± 0.05 ^c^	−22.08 ± 0.74 ^d^
8	100	2	577.48 ± 134.93 ^a,b^	0.69 ± 0.11 ^a^	−26.52 ± 1.79 ^a^
9	55	2	319.62 ± 13.65 ^c^	0.35 ± 0.03 ^c^	−24.24 ± 0.73 ^d^

If significant, differences between formulations were compared using a Bonferroni post hoc test (*p* < 0.05). ^a–e^ Within a column, means sharing the same superscript are not significantly different from each other (*p* > 0.05).

**Table 2:** Summary of the physicochemical characteristics of NPs formulated at each polymer concentration using selected formulation parameters. Results represent the average of *n* = 3 batches ± standard deviation.

PLGA-PEG (mg/mL)	PS ± SD	PDI ± SD	ZP ± SD
10	131.74 ± 3.67 ^c^	0.15 ± 0.04 ^c^	−25.85 ± 4.38
55	312.01 ± 21.87 ^b^	0.41 ± 0.03 ^b^	−20.97 ± 1.81
100	630.85 ± 52.50 ^a^	0.68 ± 0.13 ^a^	−24.25 ± 4.21

If significant, differences between formulations were compared using Tukey’s post hoc test (*p* < 0.05). ^a–c^ Within a column, means sharing the same superscript are not significantly different from each other (*p* > 0.05).

## Supplementary Materials

The following are available online at https://www.mdpi.com/1999-4923/12/11/1114/s1, Figure S1: Flow cytometric assessment of the uptake of the coumarin-6 fluorescent marker leached from NPs under in vitro conditions. (A) Representative fluorescence histograms of % cell count vs. log fluorescence using the FL-1 (green) channel. (B) Quantitative analysis of cells treated with coumarin-6 released under in vitro conditions (2 h at 37 °C) from NPs and fresh NPs. Geometric mean fluorescent intensity calculated using FlowJo software (*n* = 3), *** *p* < 0.001. Rel, release; UM, unimodal; TM, trimodal; BM, bimodal; Figure S2: In vitro release profile of coumarin-6 over time from PLGA-PEG NPs in HBSS at 37 °C (*n* = 3).
