# Supplementary material for: Optimising PLGA-PEG Nanoparticle Size and Distribution for Enhanced Drug Targeting to the Inflamed Intestinal Barrier
Source: Pharmaceutics. 2020 Nov 19;12(11):1114. doi: 10.3390/pharmaceutics12111114 (PMC7699526; doi:10.3390/pharmaceutics12111114)
Supplement: Supplementary file 1 [file pharmaceutics-12-01114-s001.pdf]

Supplement

Optimising PLGA-PEG Nanoparticle Size and Distribution for Enhanced Drug Targeting to the Inflamed Intestinal Barrier

Lauren J. Mohan, Lauren McDonald, Jacqueline S. Daly and Zebunnissa Ramtoola

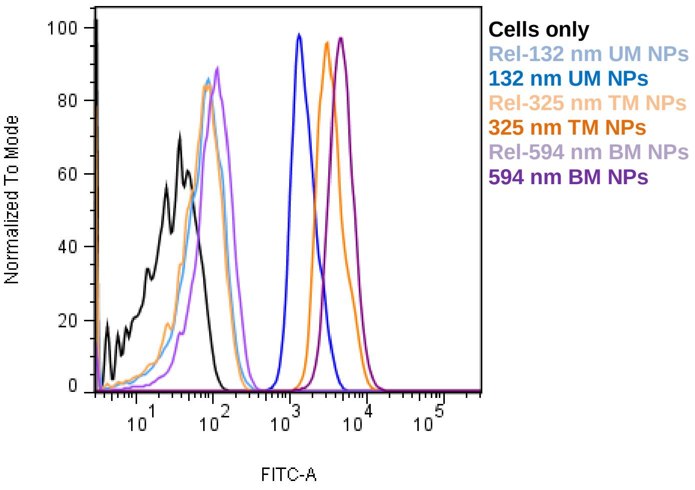

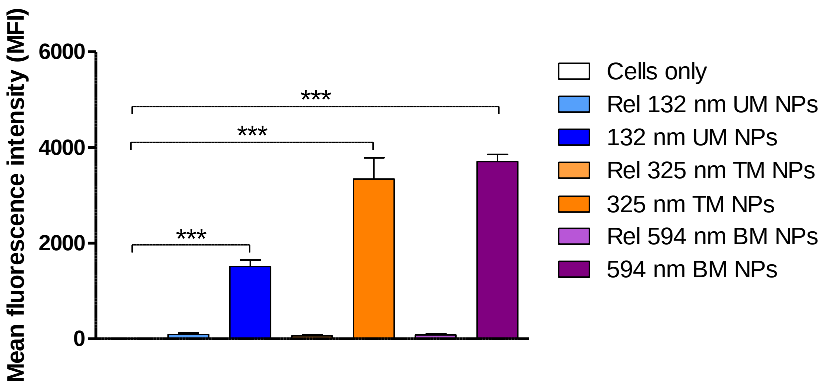

Figure S1. Flow cytometric assessment of uptake of coumarin-6 fluorescent marker leached from NPs under in vitro conditions. (**A**) Representative fluorescence histograms of % cell count vs. log fluorescence using the FL-1 (green) channel. (**B**) Quantitative analysis of cells treated with coumarin-6 released under in vitro conditions (2 h at 37 °C) from NPs and fresh NPs. Geometric mean fluorescent intensity calculated using FlowJo software (n = 3), *** p < 0.001. Rel, release; UM, unimodal; TM, trimodal; BM, bimodal.

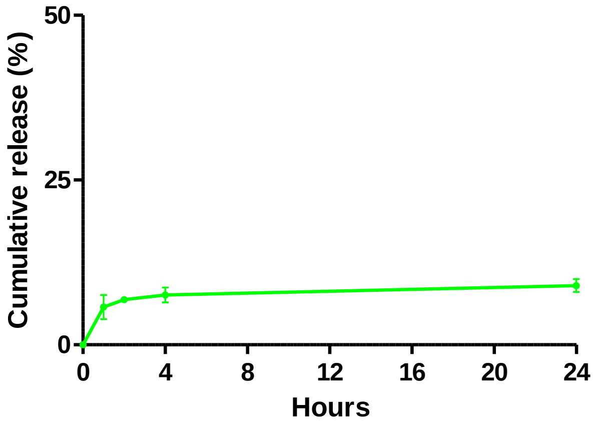

Figure S2. In vitro release profile of coumarin-6 over time from PLGA-PEG NPs in HBSS at 37 °C (*n* = 3).
